# Ten simple rules for suggesting reviewers during a paper submission

**DOI:** 10.1371/journal.pcbi.1014780

**Published:** 2026-09-16

**Authors:** Russell Schwartz, Scott Markel, Patricia M. Palagi, B. F. Francis Ouellette

**Affiliations:** 1 Ray and Stephanie Lane Computational Biology Department, Carnegie Mellon University, Pittsburgh, Pennsylvania, United States of America; 2 BIOVIA, Dassault Systèmes, San Diego, California, United States of America; 3 SIB Swiss Institute of Bioinformatics, Lausanne, Switzerland; 4 Oxford University Press, Montréal, Québec, Canada; Johns Hopkins University, UNITED STATES OF AMERICA

## Introduction

**Fig 1:**
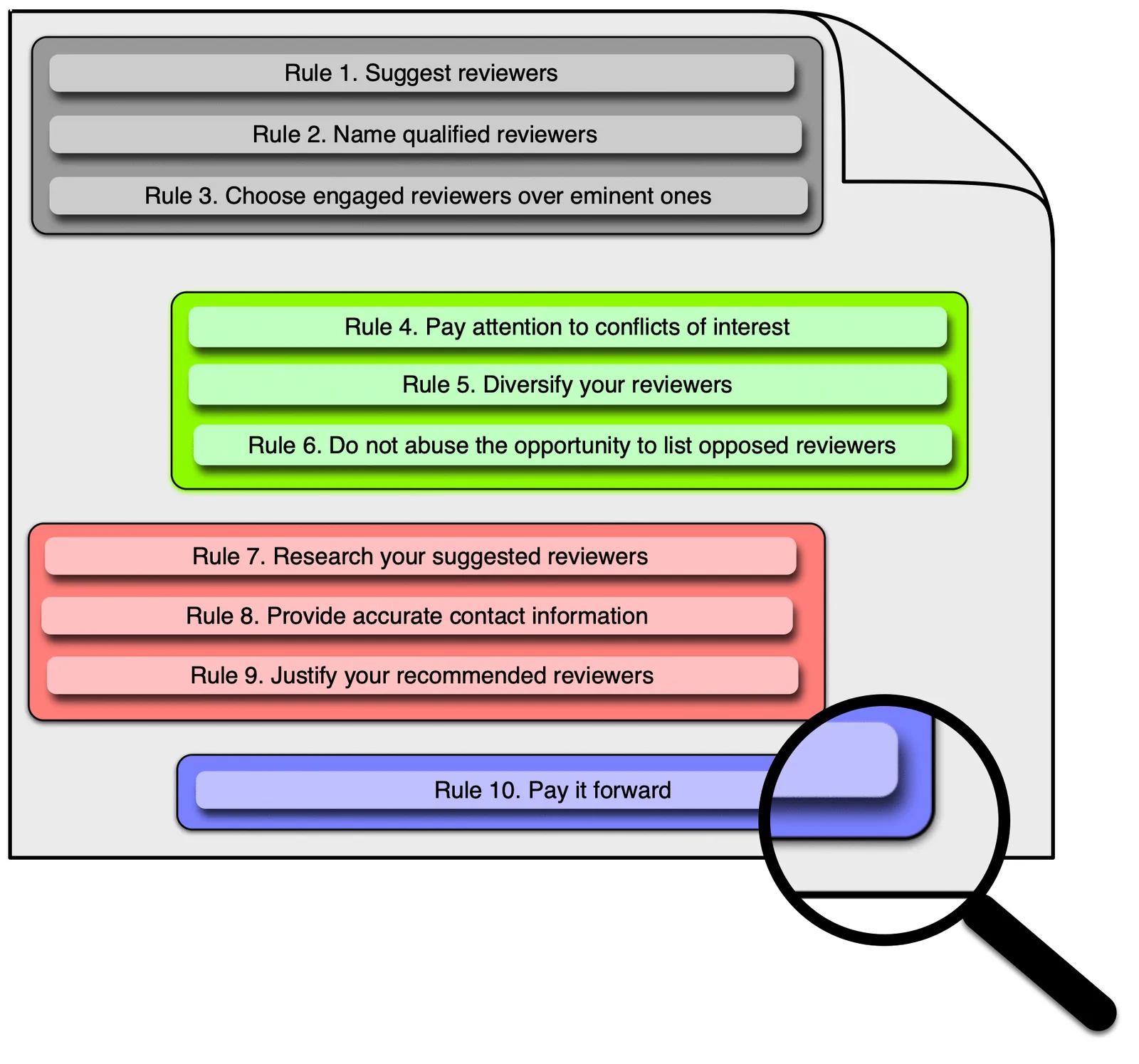
Visual summary of the ten simple rules for recommending reviewers during a paper submission. Rules are organized into four topic-based subgroups, emphasizing how an aspiring author can identify a candidate list of reviewers, refine it before submission, support the editors in making their choices, and contribute their part to the process for other authors.

This paper is intended as a guide to a small but critical part of the publication process, a way to promote the efficient review and publication of your manuscripts: recommending reviewers for a submitted paper. Not every journal allows or encourages authors to suggest reviewers, but it is a valuable contribution to the submission that warrants authors’ attention. Having the right reviewers for your paper can make all the difference in ensuring the work’s significance is appreciated, as well as in identifying key weaknesses or opportunities for improvement before publication. Editors are reporting increasing difficulty in finding qualified reviewers and securing their agreement to review [1–3], making it particularly valuable to provide them with as much useful guidance as possible. We (the editors) typically have a variety of tools at our disposal for identifying prospective reviewers, as well as our own editing experience and knowledge of the field. Yet they almost certainly do not know as much as you do about the particular specialty in which you (the authors) work, and it is mutually beneficial for you to help them navigate it. This manuscript is a guide, from the perspective of a few experienced editors and reviewers, on how an aspiring author can make the most of the process. This is not meant to diminish ethical concerns about the review process or the practice of allowing recommended reviewers [4, 5], but rather to suggest how authors can make best use of the process when it is allowed. This is in contrast to guidelines typically provided to editors for making such choices (e.g., [6]) and, we believe, complementary to prior sources of advice to authors on reviewer suggestions [7]. We are all Section Editors for *PLOS Computational Biology* and specialize in Education and “Ten Simple Rules” submissions, which have already provided guidance to readers on many aspects of scientific publishing (c.f [8–11]). The rules we present here (summarized in Fig 1) are intended to apply broadly to scientific publishing, although we recognize that they may need to be adapted to different disciplines, publication types, or societal contexts.

## Rule 1. Suggest reviewers

Most editors will want and need to find reviewers who are best suited to evaluate your submission. Finding the best reviewer is part of the editors’ responsibilities. Editors will appreciate your help with this task, and it will help your paper be reviewed in a fair and expeditious way. Provide at least the minimum number of recommended reviewers requested and more if possible. We recommend providing five or six suggested reviewers to give us flexibility in choosing the combination of expertise we want and to allow for some margin of error for reviewers who decline the invitation [12]. Remember that most requested reviewers decline, even when they are well chosen [2], so we usually need to invite several reviewers for each review we secure. *PLOS Computational Biology*, for example, has a 33% success rate in inviting reviewers. More broadly, the number of reviewer invitees who agree to review and complete reviews has steadily declined across the academic peer-review landscape [3].

## Rule 2. Name qualified reviewers

An editor will want to ensure that the people you request are qualified to review your paper. As editors, we will review the suggested reviewer’s recent publications to judge their suitability. With limited exceptions, we will not invite an author-suggested reviewer who is still a trainee (student or postdoc) with a very limited publication history, whose publications do not have some clear relationship to the subject matter of your submission, or whose publication history cannot be found online. Note, however, that we make exceptions when a trainee’s qualifications are known to us, and we also welcome suggestions from invited reviewers who can recommend a senior student or postdoctoral fellow from their own lab to co-review under their supervision.

## Rule 3. Choose engaged reviewers over eminent ones

A common mistake in naming reviewers is listing the most prominent researchers in one’s area. We sometimes try to reach out to these people, but our experience is that the success rate is nearly zero. However, we want reviewers who are technically competent, honest, and conscientious, rather than the most well-known researchers in the area. An ideal reviewer is someone who is actively publishing in the same area as the paper but not so well known that they are fielding numerous review requests and turning down almost all of them. We would much rather you point us to a newly hired assistant professor or mid-career scientist in your area who is active in the field and well-versed in current work than an established senior scientist who may be inundated with review requests and will almost certainly decline our invitation. Authors of papers you reference are often worth considering as possible reviewers.

## Rule 4. Pay attention to conflicts of interest

Several prior studies have concluded that author-recommended reviewers provide reviews of similar quality to editor-chosen reviewers but are notably more likely to give favorable recommendations [4, 13, 14]. Editors want to take advantage of your insight into who is competent to review your paper, but will be wary about the risks it creates of unfairly biasing the review process. Editors will assess potential reviewers for apparent conflicts of interest, for example, that a reviewer you suggest is at the same institution as you or has published with you in the recent past. We may lack sufficient information to identify specific conflicts, though, such as an active collaboration that has not yet led to joint publications, a business relationship, or a close personal friendship that is not apparent to us. Suggesting a reviewer with a conflict of interest with you will generally delay your paper’s review, and those delays will be larger if the reviewer must disclose the conflict than if we find it ourselves, and even larger if we discover it late in the review process and need to cancel a review and find another independent opinion. In rare cases, an undisclosed conflict of interest can even lead to the retraction of a paper’s acceptance [15, 16]. We recognize that some fields are small and tight-knit — you might consider most of those qualified to review your paper friends or at least acquaintances — and that is generally fine. However, you are expected to be aware of when it would be unethical for a given individual to review your manuscript. When in doubt, leave them out, or at least ask the journal staff for guidance.

## Rule 5. Diversify your reviewers

As editors, we are immediately suspicious of reviewer requests when all reviewers are from the same geographic location, especially if the authors share the same location. Similarly, it raises concerns if all the suggested reviewers have a history of collaborating or came from the same research group. It may be tempting to request people who are likely to know you, but we want people who will understand the work and have no conflicts of interest, which are often at odds. We understand that connections between authors and reviewers can be hard to avoid, especially in niche areas, but be mindful that they raise concerns for us as editors. We may not use your suggestions at all if we are not confident they will provide unbiased reviews. Furthermore, good editors will want a broad perspective on your paper’s merit and are less likely to use your suggestions if they do not seem to be inclusive of the broader research community in your discipline. A report from *PLOS Biology* found that reviewers from developing regions contribute a disproportionately small fraction of reviews relative to their scientific output [17], pointing to both an undertapped pool of qualified reviewers and a source of bias in the review process that good editors will want to avoid [18].

## Rule 6. Do not abuse the opportunity to list “opposed reviewers”

Another potential issue for editors is when an author provides a lengthy list of reviewers they oppose, especially if it is a “who’s who” of the paper’s research area. It is acceptable to oppose a reviewer because you want to let us know they have a conflict of interest or because they have previously demonstrated undue bias against you. It is not appropriate to oppose reviewers just because they work in the same area as you and may be publishing works you see as in competition with yours, or because they have given you a justifiably negative review in the past. Other people doing state-of-the-art work on questions similar to yours are precisely who we want as reviewers. Including opposing reviewers is not required. Also bear in mind that while editors prefer not to invite a reviewer who is listed as opposed, they reserve the right to do so if they believe the reviewer will provide a fair and scientifically important review.

## Rule 7. Research your suggested reviewers

A poor choice of reviewer wastes your time and the editor’s. A poor choice might be a reviewer who proves unreachable, who declines to review, who is unqualified and provides an uninformative review, or agrees to review but then fails to deliver. Even the best-chosen reviewer may say “no” and one cannot always anticipate this, but it is to your advantage to avoid obvious reasons a reviewer will decline or fail to provide a useful review. For example, we recommend verifying that the reviewer is still publishing in your paper’s field and remains affiliated with the institution you provided. These are generally small things for you to check, and will avoid the editor wasting time pursuing a reviewer who will not help move your paper to publication.

## Rule 8. Provide accurate contact information

People change jobs from time to time, and there is a high chance that a qualified reviewer is already registered in our reviewing system with out-of-date contact information. We often do not realize that until an email invitation bounces (or worse, gets no reply). Given the volume of invitations we handle as editors, we will not spend much time tracking down a reviewer if the correct contact information is not readily available online. If you feel someone will review your paper well, it is to your advantage to make sure we can find them easily.

## Rule 9. Justify your recommended reviewers

A reviewer recommendation will often come with an opportunity to provide a brief explanation of why you chose that reviewer. This helps us prioritize reviewers and allays our concerns about whether a particular reviewer is suitable for your paper. An explanation need not be long; one sentence telling us what achievements or expertise they have relevant to your submission is sufficient. If the automated submission system does not provide you with an opportunity to justify reviewers when you suggest them, you might also put that in your cover letter.

## Rule 10. Pay it forward

A rough estimate is that for every paper you publish, two to three reviewers will have reviewed the final version of your paper that was accepted, and perhaps two to three times that many will have reviewed some version of the paper if it was submitted and rejected elsewhere before the final accepted version [2]. Scientific publishing is a system that depends on volunteers, particularly those who write papers, and requires a proportionate share of reviewing others’ papers. We can calibrate that by the fact that a paper typically has multiple authors, but you should be reviewing several times as many papers as you publish to be carrying your fair share of the community’s reviewing burden. We volunteer our time as editors and reviewers of many manuscripts, in addition to our other duties as scientists, educators, and administrators. We are all busy, but no one who has the time to publish should be too busy or too important to do their share of reviewing. If you have to decline a reviewing invitation, reply quickly and suggest two or three alternatives.

## Discussion

Reviewing is crucial to the publication process. We, as editors, share a common interest with authors in finding qualified reviewers and moving your paper expeditiously through the reviewing process. Recommending reviewers is a vital step in accomplishing this. The rules provided in this paper are intended to guide authors in helping us help YOU get your paper reviewed effectively and efficiently. The publishing landscape is becoming increasingly challenging as the number of papers and authors grows [2]. We, as editors, rely more than ever on authors to help us find good reviewers and avoid wasting time on those who will decline to review, give poor-quality reviews, or be excluded for conflicts of interest. There are many uncertainties in where scientific publishing is going as an industry. For example, it will grapple with larger numbers of authors in our disciplines [19], ongoing concerns about reproducibility [20], and emerging questions about the potential uses and misuses of artificial intelligence in writing and reviewing [21, 22]. Nonetheless, we predict that these trends will only increase the need to identify reliable authorities for review and to help authors make the best use of their opportunities to support that process.
